# Exploring the protective mechanism of baicalin in treatment of atherosclerosis using endothelial cells deregulation model and network pharmacology

**DOI:** 10.1186/s12906-022-03738-3

**Published:** 2022-10-03

**Authors:** Mingshuang Li, Conglin Ren

**Affiliations:** 1 Taizhou Hospital, Shanghai University of Traditional Chinese Medicine, Taizhou, Zhejiang China; 2 Taizhou Hospital of Traditional Chinese Medicine, Taizhou, Zhejiang China

**Keywords:** Atherosclerosis, Baicalin, Network Pharmacology, Oxidative stress, NOX4

## Abstract

**Background:**

Baicalin is a generally available flavonoid with potent biological activity. The present study aimed to assess the underlying mechanism of baicalin in treatment of atherosclerosis (AS) with the help of network pharmacology, molecular docking and experimental validation.

**Methods:**

The target genes of baicalin and AS were identified from public databases, and the overlapping results were considered to be baicalin-AS targets. Core target genes of baicalin were obtained through the PPI network and validated by a clinical microarray dataset (GSE132651). Human aortic endothelial cells (HAECs) were treated with Lipopolysaccharide (LPS) to construct an endothelial injury model. The expression of NOX4 was examined by real-time qPCR and western blot. Flow cytometry was used to detect intracellular levels of reactive oxygen species (ROS). Furthermore, HAECs were transfected with NOX4-specific siRNA and then co-stimulated with baicalin and LPS to investigate whether NOX4 was involved in the anti-oxidative stress effects of baicalin.

**Results:**

In this study, baicalin had 45 biological targets against AS. Functional enrichment analysis demonstrated that most targets were involved in oxidative stress. Using the CytoHubba plug-in, we obtained the top 10 genes in the PPI network ranked by the EPC algorithm. Molecular docking and microarray dataset validation indicated that NOX4 may be an essential target of baicalin, and its expression was significantly suppressed in AS samples compared to controls. In endothelial injury model, intervention of HAECs with baicalin increased the expression levels of NOX4 and NOS3 (eNOS), and decreased LPS-induced ROS generation. After inhibition of NOX4, the anti-ROS-generating effect of baicalin was abolished.

**Conclusion:**

Collectively, we combined network pharmacology and endothelial injury models to investigate the anti-AS mechanism of baicalin. The results demonstrate that baicalin may exert anti-oxidative stress effects by targeting NOX4, providing new mechanisms and insights to baicalin for the treatment of AS.

**Supplementary Information:**

The online version contains supplementary material available at 10.1186/s12906-022-03738-3.

## Background

Atherosclerosis (AS), the leading cause of cardiovascular disease (CVD), is difficult to detect in early stages until lipid-burdened plaque ruptures or erodes, eventually leading to thrombosis and tissue damage [1]. Endothelial cells (ECs) are the first line of defense for vascular health. Both activation of ECs and infiltration of circulating monocytes are crucial factors in occurrence and progression of AS. Inflammatory ECs recruit leukocytes by expressing adhesion molecules; circulating monocytes infiltrate into damaged endothelium and differentiate into macrophages, which are further transformed into foam cells in response to excessive lipid load. This process promotes the release of pro-inflammatory factors and generation of reactive oxygen species (ROS) [2]. ROS, mainly originating from the NADPH oxidase (NOX) complex, mediates oxidative stress that promotes the progression of AS by inducing inflammation, apoptosis and alterations in vascular tone [3]. Increased expression of NOX1, NOX2 and NOX5 has been reported as contributing to AS and vascular diseases [4, 5]. However, NOX4 is a protective gene in ECs that exerts anti-oxidative effects by increasing the release of NO through AKT-dependent phosphorylation [6, 7].

Chinese herbal medicine is the most widely used form of complementary and alternative medicine, which has been practiced for centuries in Asia. In treatment of coronavirus disease 2019, the effects were remarkable [8–11]. Studies on the components of herbal medicine have shown that baicalin is mainly found in the roots of Scutellaria baicalensis, but also in Salvia miltiorrhiza, Platycodon grandiflorum, Paeonia lactiflora and Sempervivum [12–15]. As a representative of the flavonoids, baicalin has been shown to have a variety of pharmacological functions, such as reducing oxidative stress and inflammation through the NF-κB pathway, blocking the growth cycle of vascular smooth muscle cells, and inhibiting intimal proliferation after injury [16].

**Fig. 1:**
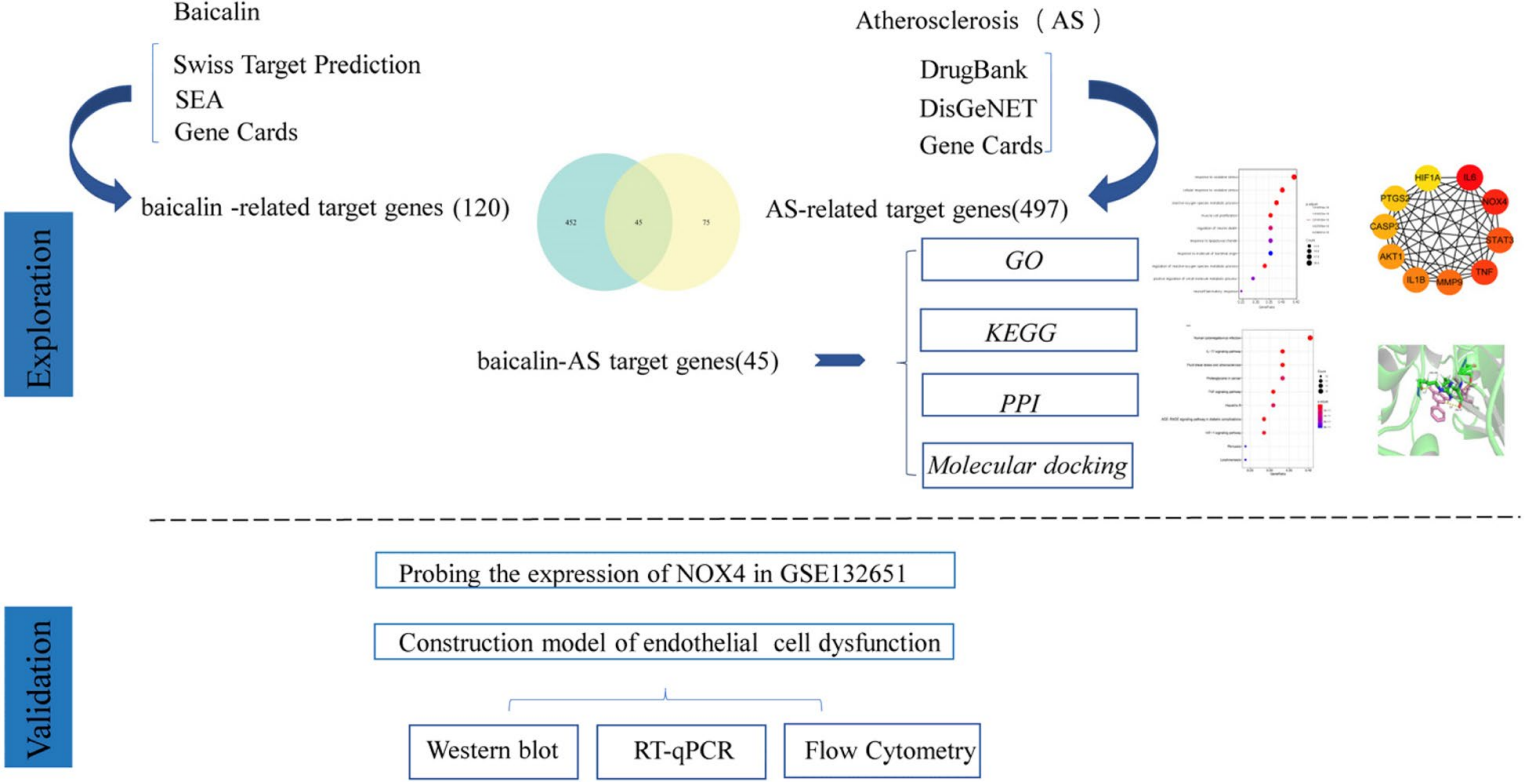
The flowchart for exploring the molecular mechanisms of baicalin in treatment of AS using network pharmacology and experimental validation. AS: atherosclerosis

Network pharmacology, first proposed by Andrewl Hopkins in 2007 [17], combines pharmacology with bioinformatics to reveal specific targets for drug interventions in disease process, helping to advance the development of precision medicine [18]. It also can be used to analyze the mechanism of herbal formula, such as BuYangHuanWu decoction [19] and Chaihu Lizhong Tang [20]. In current study, we used multiple public databases to predict baicalin-AS targets and set up pharmacological network. Through molecular docking and microarray dataset (GSE132651) validation, we hypothesized that NOX4 might be an essential target of baicalin in treatment of AS. Thereafter, endothelial injury model and NOX4-specific siRNA were constructed to investigate whether NOX4 was involved in the anti-oxidative stress effects of baicalin. The flow chart was exhibited in Fig. 1.

## Methods

### Predicting target genes of baicalin

Firstly, we obtained the pharmacokinetics parameters of baicalin by TCMSP database [21]. The parameters (oral bioavailability = 40.12%, druglike = 0.75) indicated that baicalin has positive pharmacodynamic effects [22]. Following this, further predictions including absorption, distribution, metabolism, excretion and toxicity (ADMET) were performed using the admetSAR 2.0 server regression model [23]. The prediction results were presented in Supplementary Files (Table S1). Subsequently, we queried the PubChem database (https://pubchem.ncbi.nlm.nih.gov/) for the 2D structures, molecular formulas, and canonical SMILES strings. SDF file of baicalin was entered into three public databases for predicting potential targets including Similarity ensemble approach (SEA) [24], Swiss Target Prediction [25]. and Gene Cards [26]. In the SEA database, the top 100 results ranked by *P*-value were retained; for the Swiss Target Prediction, results with probability greater than 0.1 were selected; while among the Gene cards, those with score greater than 0.1 were reserved. For each database, species were restricted to ‘Homo sapiens’. After pooling the results, only genes predicted by two or three databases at the same time were screened as high confidence target genes.

### Collection of target genes for AS

Similarly, to identify key genes associated with the development of AS, we searched for the keyword “atherosclerosis” in multiple databases(i.e., DisGeNET [27], Gene Cards and DrugBank [28]) and collected overlapping results as vital pathogenic targets of AS for further study.

### Identification of baicalin-AS targets

The target genes of baicalin were matched with AS-related targets, and overlapping data were considered to be potential targets of baicalin for AS treatment. Venn diagrams were generated in R software (version 3.6.2) using “VennDiagram” package to visualize the number of intersecting genes.

### Functional enrichment analysis of baicalin-AS targets

Gene Ontology (GO) and KEGG can identify enriched biological functions and pathways [29–32]. To obtain functional annotation information of baicalin-AS targets, we performed Gene Ontology (GO) and KEGG analysis by R packages including “clusterProfiler” [33] and “ggplot2” [34]. The results of enrichment analysis with statistical significance (adjusted *p*-value < 0.05) were screened and then visualized by bubble plots and multi-level interaction networks.

### Protein–protein interaction (PPI) analysis and central module identification

The STRING database (https://string-db.org/, version 10.5) [35, 36] was used to build PPI network of baicalin-AS targets. To make the network more concise and with higher confidence, we kept nodes and edges with interaction score greater than 0.4 and hid the disconnected nodes. Thereafter, the PPI information was aggregated into a “TSV” format file and imported into Cytoscape software (version 3.8) [37] for visualization. The Edge Percolation Component (EPC) algorithm of the Cytohubba plug-in in Cytoscape was applied to calculate the score of each node and determine the top 10 hub genes based on their ranking.

### Molecular docking

The SDF file for baicalin was downloaded from the PubChem database and converted to PDB format by OpenBabel. The 3D crystal structures of the five hub target genes were downloaded from the RCSB Protein Data Bank (http://www.pdb.org/). The ligand and receptor files were imported into AutoDock Tools 1.5.6 and prepared for docking, including dehydration and hydrogenation, which were exported and saved in PDBQT format. Then, the appropriate parameters were set and the ‘Grid box’ was set to maximum for blind docking. A binding energy of less than -5 kJ∙mol-1 indicates that the ligand can spontaneously bind to the receptor. Docking results were visualized using PyMOL.

### Probing the expression of hub genes in endothelial injury model

To examine the gene expression patterns (up- or down-regulation) of hub genes in endothelial injury model, we selected GSE132651 dataset, a microarray study that quantifies gene expression of ECs in living subjects, for further analysis. This dataset consisted of 19 samples, including 6 normal controls and 13 subjects with abnormal endothelial function (showing a risk for early AS). We retrieved original matrix files from GEO repository (https://www.ncbi.nlm.nih.gov/geo/), filtered the mRNA expression levels of hub genes between two groups using “Limma” package [38] and visualized by GraphPad Prism 7.0.

### Reagents

Baicalin (purity > 98%) was supplied by Yuan-ye biotechnology (Shanghai, China). Lipopolysaccharide (LPS), DMSO, and cell counting kit-8 (CCK-8) were obtained from Meilun biology (Dalian, China). EGM-2 Bullet Kit were obtained from Lonza (#CC-3162, Basel, Switzerland), including endothelial cell basal medium, FBS, VEGF, etc. RNA-quick purification kit was purchased from Yi-shan biotechnology (Shanghai, China). First strand cDNA synthesis kit and SYBR Green master mix were obtained from Thermo Scientific (Shanghai, China). PCR primers were synthesized by Sangon biotechnology (Shanghai, China). DCFDA/H2DCFDA cellular ROS assay kit was obtained from Abcam (Massachusetts, USA).

### Cell culture

Human aortic endothelial cells (HAECs) were purchased from Chinese Tissue Culture Collections (CTCC). Cells were cultured in MEM medium (supplemented with 2% FBS, 2 mM L-glutamine, 1000 units/mL penicillin, 100 μg/mL streptomycin, and 0.25 μg/mL amphotericin B) and maintained in a humidified incubator at 37℃ with 5% CO2. For experimental group, cells were pretreated with optimal concentrations of baicalin for 12 h, and then stimulated with LPS (1 μg/mL) for 6 h.

### Cytotoxicity assay of baicalin

The cytotoxicity of baicalin was tested by CCK-8 assay according to the manufacturer’s instructions. Briefly, to obtain optimal dose of intervention, HAECs (8 × 10^4^/ml) were seeded in 96-well culture plates and exposed to gradually increasing concentrations (0-400 μM) of baicalin for 12 h. The absorbance value of each well was measured at 450 nm using EnSpire multifunctional microplate reader (PerkinElmer, USA). Cell viability at different concentrations was calculated from the absorbance of the blank (without HAECs) and control (baicalin = 0 μM) wells.

### Reverse transcription quantitative PCR (RT-qPCR)

Total RNA was extracted from HAECs and its purity was checked using Nanodrop (Thermo Scientific, USA). After that, mRNA was transcribed into the first-strand cDNA and subjected to polymerase chain reaction on 7500 real-time PCR system (Applied Biosystems, USA). We chose GAPDH as endogenous control and compared gene expression based on the 2^—△△CT^ algorithm. The primer sequences used in current study were listed in Table 1.

**Table 1:** RT-qPCR primers used in this study

Gene name	Primer sequences
GAPDH	forward 5′-GTCTCCTCTGACTTCAACAGCG-3′
	reverse 5′-ACCACCCTGTTGCTGTAGCCAA-3′
NOX4	forward 5′-GCCAGAGTATCACTACCTCCAC-3′
	reverse 5′-GTGACTCCTCAAATGGGCTTCC-3′
NOS3 (eNOS)	forward 5′-GAAGGCGACAATCCTGTATGGC-3′
	reverse 5′-TGTTCGAGGGACACCACGTCAT-3′

### Western blot

Total proteins were extracted from HAECs using RIPA lysis buffer (Beyotime Biotechnology, China), followed by concentration detection with the BCA protein assay kit (Sangon Biotech, China). Equal amounts of protein were separated by 10% SDS-PAGE and transferred onto PVDF membranes. Afterwards, PVDF membranes were blocked with 5% skim milk for 1 h and then incubated with primary antibodies including β-actin (1:1000, Cell Signaling Technology (CST), #3700), NOX4(1:1000, abcam, #ab133303) at 4 °C overnight. The membranes were incubated with secondary antibodies (Beyotime Biotechnology, Shanghai, China) for 2 h. Differences in gray values were calculated using the Image Lab software (Bio-Rad Laboratories Inc). The experiment was repeated three times independently.

### Transfection

For small interfering RNA (siRNA) transfection, HAECs were transfected with NOX4-specific siRNA (Tsingke Biotechnology, Beijing, China) or negative control siRNA (Table S2) for 6 h by Lipofectamine 2000 (Invitrogen, Carlsbad, CA, USA) with Opti-MEM Reduced-Serum Medium (Gibco). Then, the medium was replaced with regular culture medium containing serum and the cells were cultured for 12 h. Next, the cells were exposed to LPS (1 μg/mL) with or without baicalin. RT-qPCR and western blot analysis were used to evaluate the efficiency of siRNA.

### Intracellular ROS measurement

Briefly, HAECs pretreated with baicalin for 12 h were harvested and stained with 20 μM DCFDA in culture medium. After incubation at 37℃ for 30 min (protected from light), the cells were washed with 1X buffer and treated with 1 μg/ml LPS for 6 h. Then, cells were gently pipetted to make single cell suspension and analyzed on flow cytometer (BD Accuri C6, USA). Data processing was carried out on FlowJo software (version 10, USA).

### Statistical analysis

Statistical analysis was performed in R software or GraphPad Prism 7.0 (California, USA). When analyzed using GraphPad Prism, the data were shown as mean ± SEM. The t-test was used for comparisons between two groups; one-way analysis of variance was taken for comparisons between three or more groups. Results with *p*-value < 0.05 were considered significant.

## Results

### Collection target genes of baicalin

**Fig. 2:**
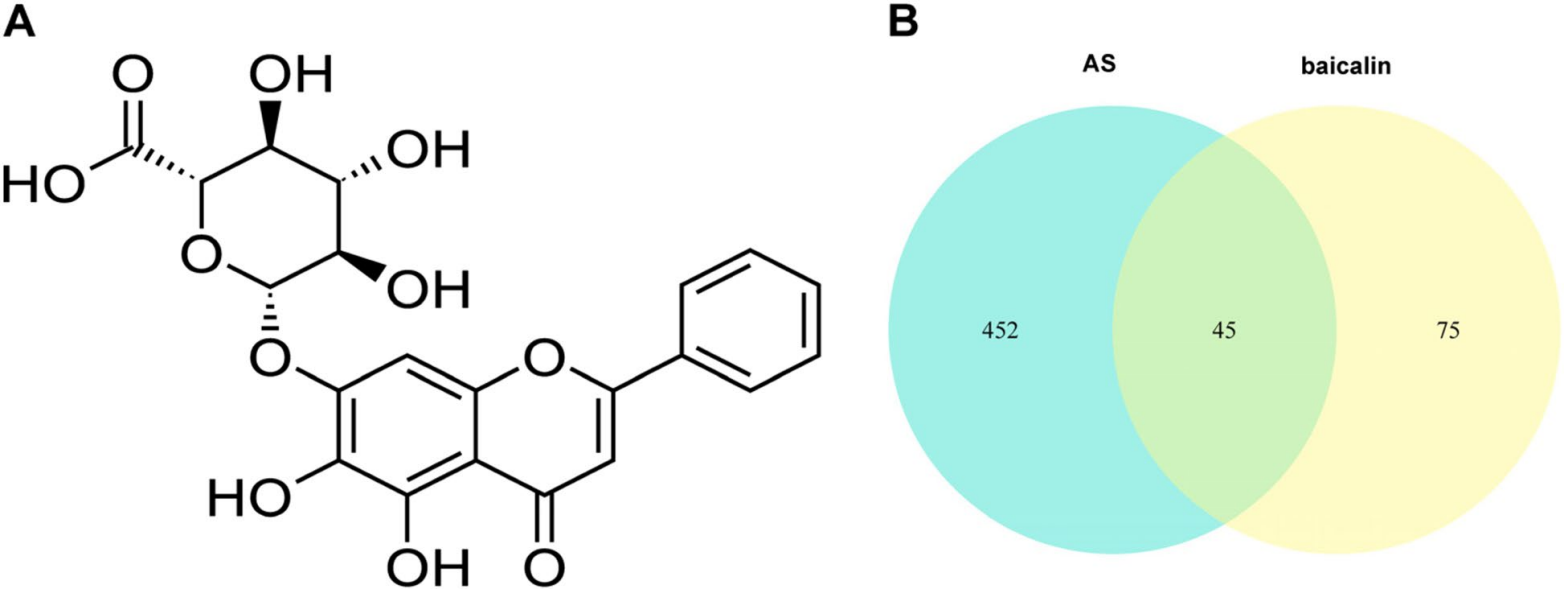
Identification of baicalin-AS target genes. **A** The chemical structure of baicalin. **B** Venn diagram showing the overlap among target genes of baicalin (blue) and AS related target genes (yellow). A total of 45 gene products were identified as common

The skeletal formula of baicalin was created using ACD/ChemSketch software (version 12.0, Advanced Chemistry Development, Toronto, Canada) (Fig. 2A). We collated the target genes for baicalin predicted by the SEA, Swiss Target Prediction and Gene Cards databases. Based on the screening criteria mentioned in “Methods”, we obtained 120 targets with high confidence for baicalin.

### Identification of AS-related target genes

By integrating disease targets derived from the DisGeNET, Gene Cards, and DrugBank databases, we ultimately retained 497 genes that appeared in at least two databases. Admittedly, the pathophysiology of AS is complicated, with an extensive number of genes involved and interacting with each other.

### Baicalin-AS target genes

We obtained intersection data by matching targes of baicalin with AS-related target genes. As shown in Venn diagram (Fig. 2B and Table S3), 45 baicalin-AS target genes were generated, which were potential targets of baicalin for AS treatment.

### Functional enrichment analysis of baicalin-AS targets

**Fig. 3:**
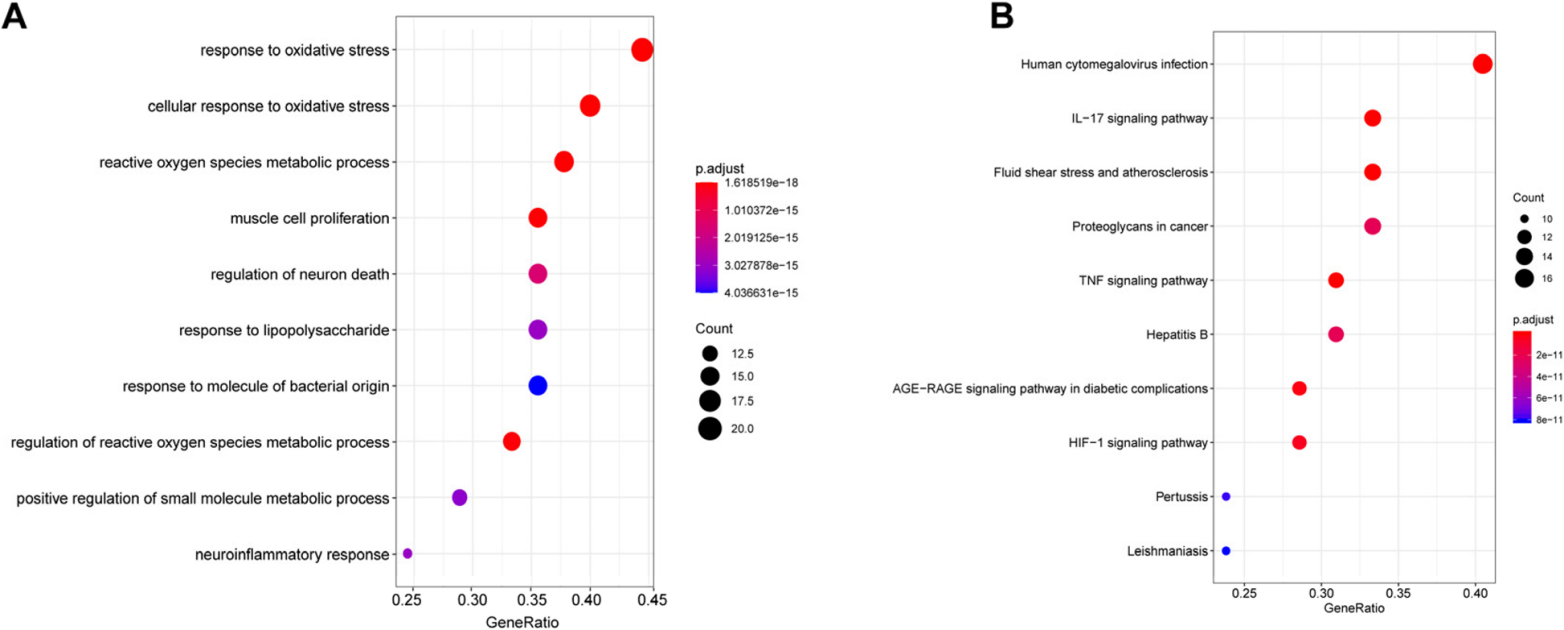
Functional enrichment analysis of baicalin-AS target genes. **A** Top 10 terms of BP enrichment analysis for baicalin-AS target genes. **B** Top 10 terms of KEGG enrichment analysis for baicalin-AS target genes

**Fig. 4:**
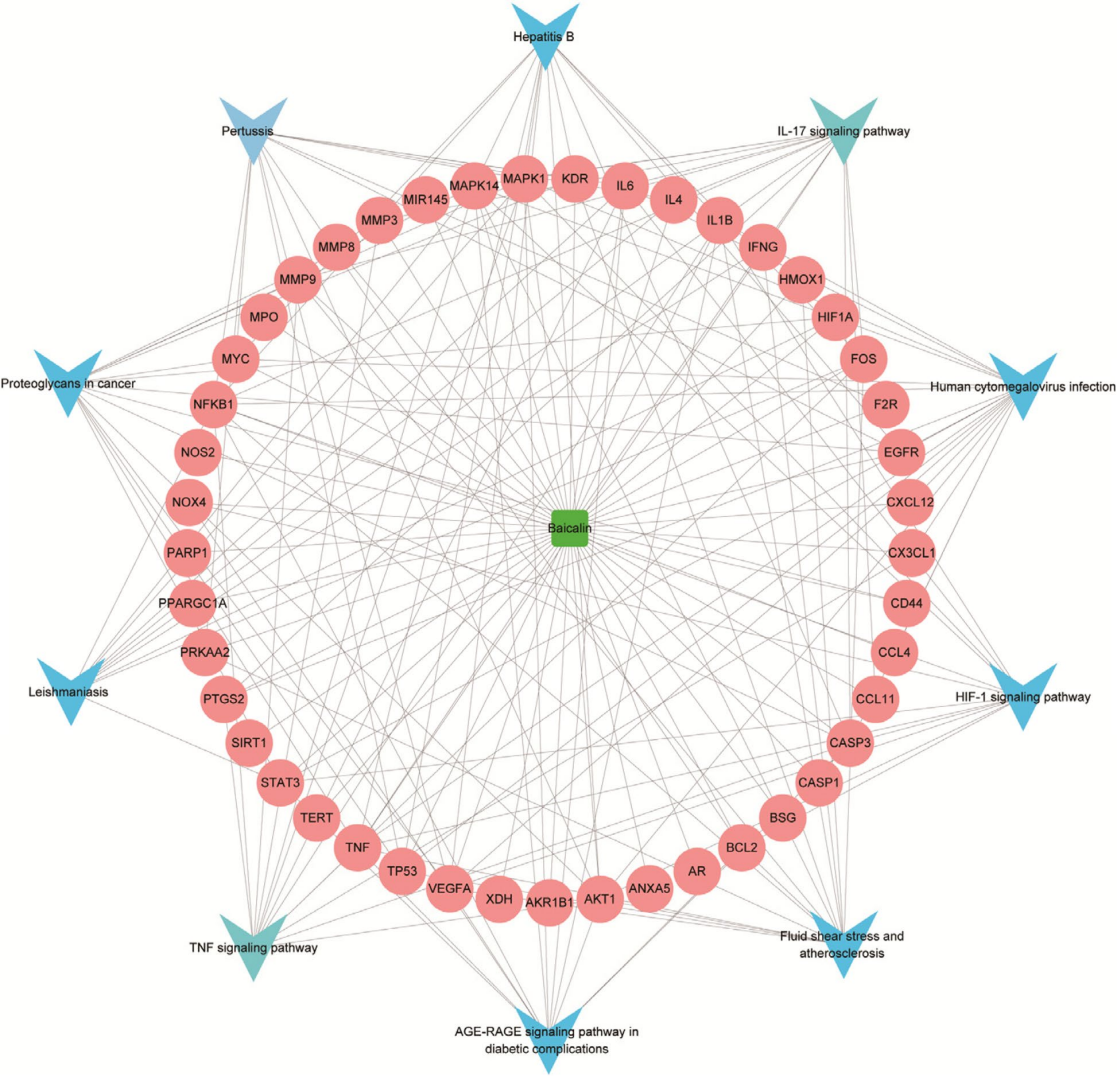
Compound-target-pathway networks(C-P–T). Green squares represented compounds and pink circles represented targets. The blue V shapes represented the pathways

The potential biological processes (BP) and signaling pathways related to baicalin-AS targets were explored through functional enrichment analysis. The top 10 possible BP items ordered by “gene ratio” were shown in Fig. 3A, including responses to oxidative stress, cellular responses to oxidative stress, metabolic processes of reactive oxygen species (ROS), etc. On the other hand, KEGG pathway analysis showed that baicalin-AS targets were mainly involved in human cytomegalovirus infection, IL-17 signaling pathway, fluid shear stress and atherosclerosis, etc. (Fig. 3B). Overall, enrichment analysis showed that most of the target genes were associated with inflammation, suggesting that baicalin may act against AS through anti-inflammatory effects. We sorted out the relationships between baicalin, target genes and signaling pathways in Excel, and then imported the information into Cytoscape software to construct a multi-level interaction network. As shown in the Fig. 4, numerous genes were involved in inflammatory and immune-related pathways. Genes involved in fluid shear stress and atherosclerosis include IL1β, TNF, MMP9, AKT1, and NOX4; genes associated with AGE-RAGE signaling pathway in diabetic complications include AKT1, IL1β, and NOX4; while genes associated with HIF signaling pathway contain STAT3, NOX4, NFκB, and MAPK1.

### PPI network construction and central module identification

**Fig. 5:**
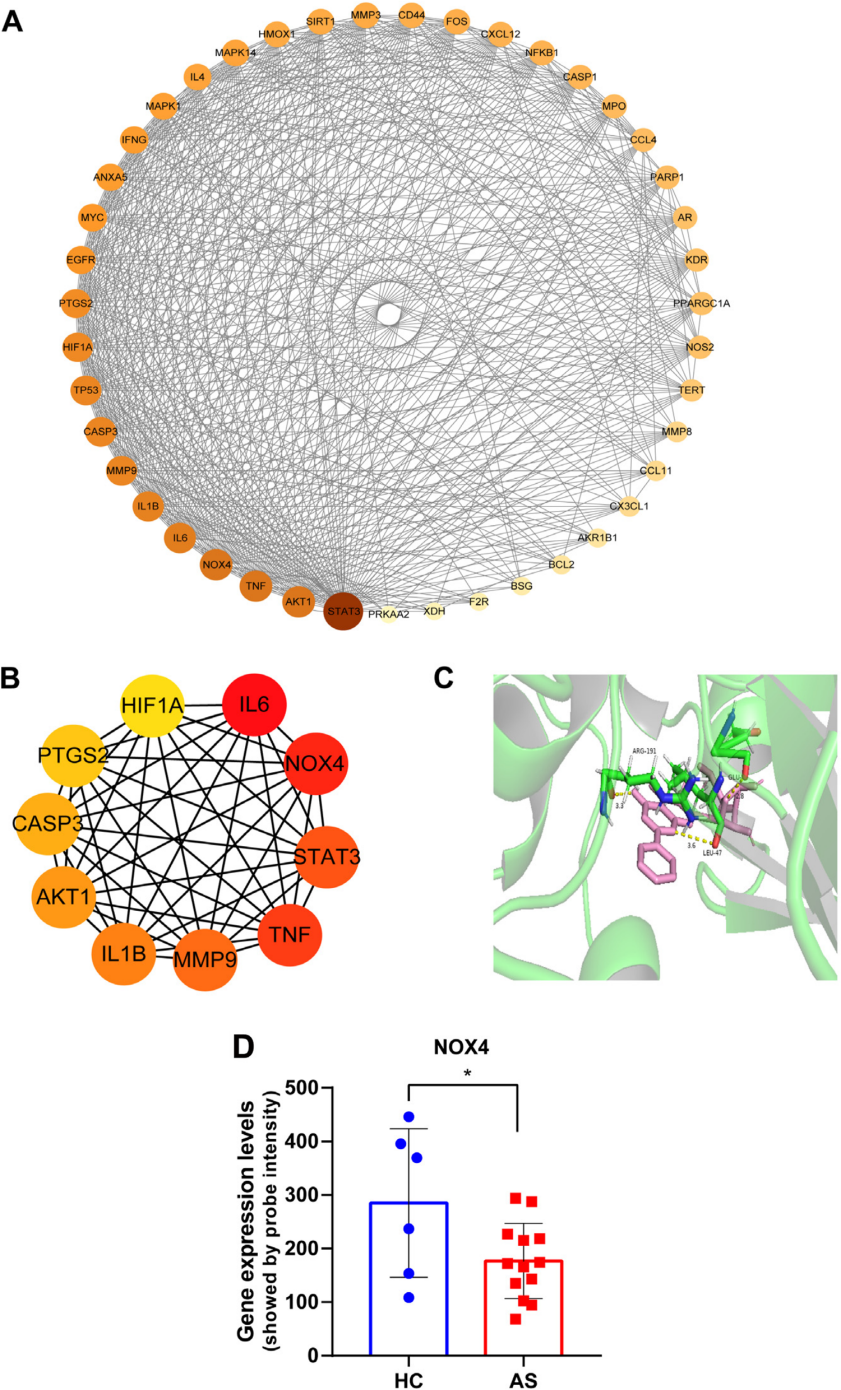
NOX4 was the core target gene of baicalin. **A** PPI network topological analysis. Based on gene intersection, this study acquired altogether 44 protein nodes and 10 hub target genes from topological analysis. As shown, the node color represented the score, with deeper and redder nodes meaning higher degree value. **B** The top 10 hub genes were screened from (A) using CytoHubba. As shown, the node color represented the score, with deeper and redder nodes meaning higher EPC scores. **C** Molecular docking of NOX4 with baicalin, affinity =  − 9.31 kcal/mol. **D** NOX4 mRNA expression in GSE132651 based on probe intensity. (*P < 0.05)

To further explore the association between the 45 target genes, we constructed a PPI network, containing 44 nodes and 542 edges, via STRING database (Fig. 5A). In parallel, the top 10 target genes (IL6, NOX4, TNF, STAT3, MMP9, IL1B, AKT1, CASP3, PTGS2 and HIF1A) were calculated using the cytoHubba plug-in and shown in Fig. 5B, which were considered to be hub target genes for baicalin in treatment of AS.

### Verification of ligand–receptor interaction

In order to select the most powerful binding molecules, we selected the top 5 target genes in the hub module for molecular docking with baicalin. It was confirmed that baicalin had a high binding activity with the hub genes, and the binding energy to NOX4 is the strongest (Table 2). Thus, it was hypothesized that NOX4 might be an essential target of baicalin in the treatment of AS (Fig. 5C).

**Table 2:** Results of molecular docking of hub target genes and baicalin

The target gene	PDB ID	Binding energies (kcal/mol)	PPI Score by EPC method
NOX4	4UT3	-9.31	21.157
IL6	4O9H	-5.26	21.15
STAT3	6NUQ	-5.73	21.15
TNF	6Q01	-6.21	20.574
MMP9	5TH6	-5.48	20.546

### NOX4 expression was down-regulated in endothelial injury condition

To further investigate the role of NOX4 in AS, we selected the GSE132651 dataset for validation. The details of gene expression matrix were shown in Supplementary Files. As illustrated in Fig. 5D, inter-group comparison showed that NOX4 expression was significantly suppressed in AS sample group (probe intensity, 176.8 ± 19.42) compared to controls (probe intensity, 285.1 ± 56.56) (*P* < 0.05).

### Cytotoxic effects of baicalin on HAECs

**Fig. 6:**
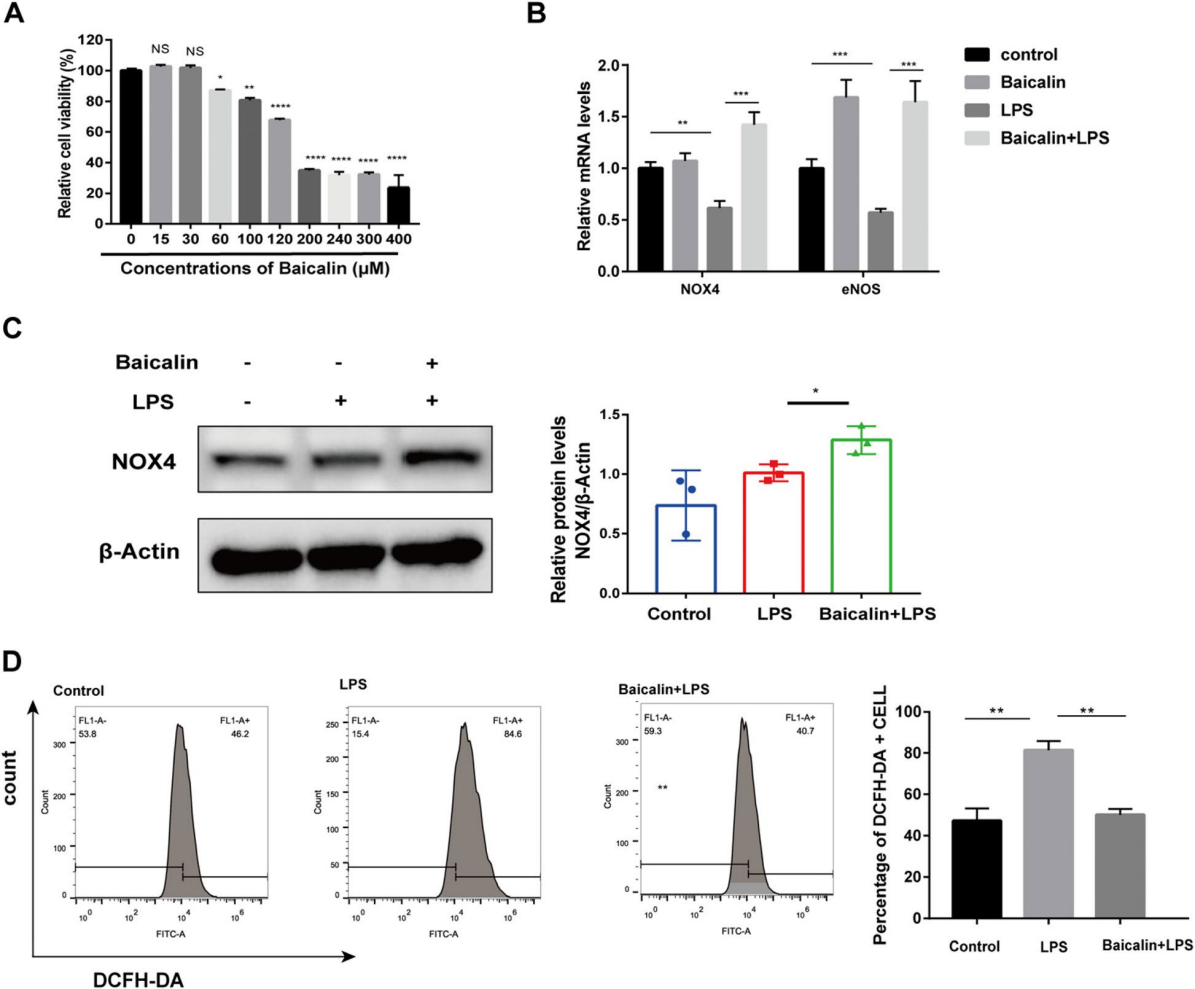
Baicalin increased the expression levels of NOX4 and decreased the LPS-induced ROS generation. **A** Effect of different concentrations of baicalin on viability of HAECs assessed by CCK8. **B** Baicalin modulated mRNA expression level of NOX4 and eNOS. GAPDH was used as the control. **C** HAECs were cultured with LPS supplemented with baicalin or not. Cell lysates were then analyzed using Western blotting assay. Each histogram value is the ratio of the scan value for NOX4 divided by the value for β-actin. The values represent mean ± SEM of three independent experiments. **D** Baicalin inhibited ROS release. HAECs were exposed to baicalin for 12 h and stained with 20 μM DCFDA for 30 min, followed by 1 μg/ml LPS for 6 h. ROS levels were detected by flow cytometry analysis. Data were shown as mean ± SEM. (*n* = 3, *P < 0.05, **P < 0.01, ***P < 0.001, ***** *P* < 0.0001)

The cytotoxic effects of multiple concentration gradients of baicalin on HAECs were examined by CCK-8 assay. The results showed that baicalin had no obvious cytotoxicity to HAECs when the treatment concentration was less than 30 μM (Fig. 6A). Therefore, we chose the maximum safe concentration (30 μM) for subsequent experiments.

### Baicalin increased NOX4 and eNOS expression in LPS-stimulated HAEC

In the GO analysis section, we noted that the enrichment of “oxidative stress” and “ROS production” was very high, suggesting that baicalin may have a modulating effect on oxidative stress and ROS. To further probe whether baicalin has a protective effect on AS, we established an LPS-induced endothelial injury model for in vitro experiments [39, 40].

NOX4 is one of the hub targets of baicalin, which can inhibit oxidative stress by promoting the production of NO [41]. NOS3 (eNOS) can interact with L-arginine to promote the physiological synthesis of nitric oxide (NO) [42]. In the LPS-induced endothelial cell dysfunction model, mRNA expression of NOX4 and eNOS was decreased compared to the control group, which was notably reversed after 12 h of treatment with baicalin. (Fig. 6B). To further demonstrate the relationship between baicalin and NOX4, we examined NOX4 protein levels by western blot, which showed that baicalin also elevated NOX4 protein levels in HAECs (Fig. 6C). These data suggested that baicalin may exert protective effects on LPS-induced HAECs by targeting NOX4 and eNOS, thereby alleviating oxidative stress levels.

### Baicalin attenuated ROS release from LPS-stimulated HAECs

Excessive intracellular ROS cause endothelial dysfunction and oxidative stress injury. In this study, intracellular ROS levels were measured by DCFH-DA staining to assess whether baicalin was involved in regulating ROS in HAECs. Flow analysis demonstrated that ROS levels were increased significantly in the LPS-stimulated group compared to the control group. And the ROS levels were down-regulated after baicalin treatment compared to the LPS-stimulated group. The results showed that baicalin could attenuate ROS release in HAECs (Fig. 6D).

### NOX4 plays an essential role in the anti-oxidative stress of baicalin

**Fig. 7:**
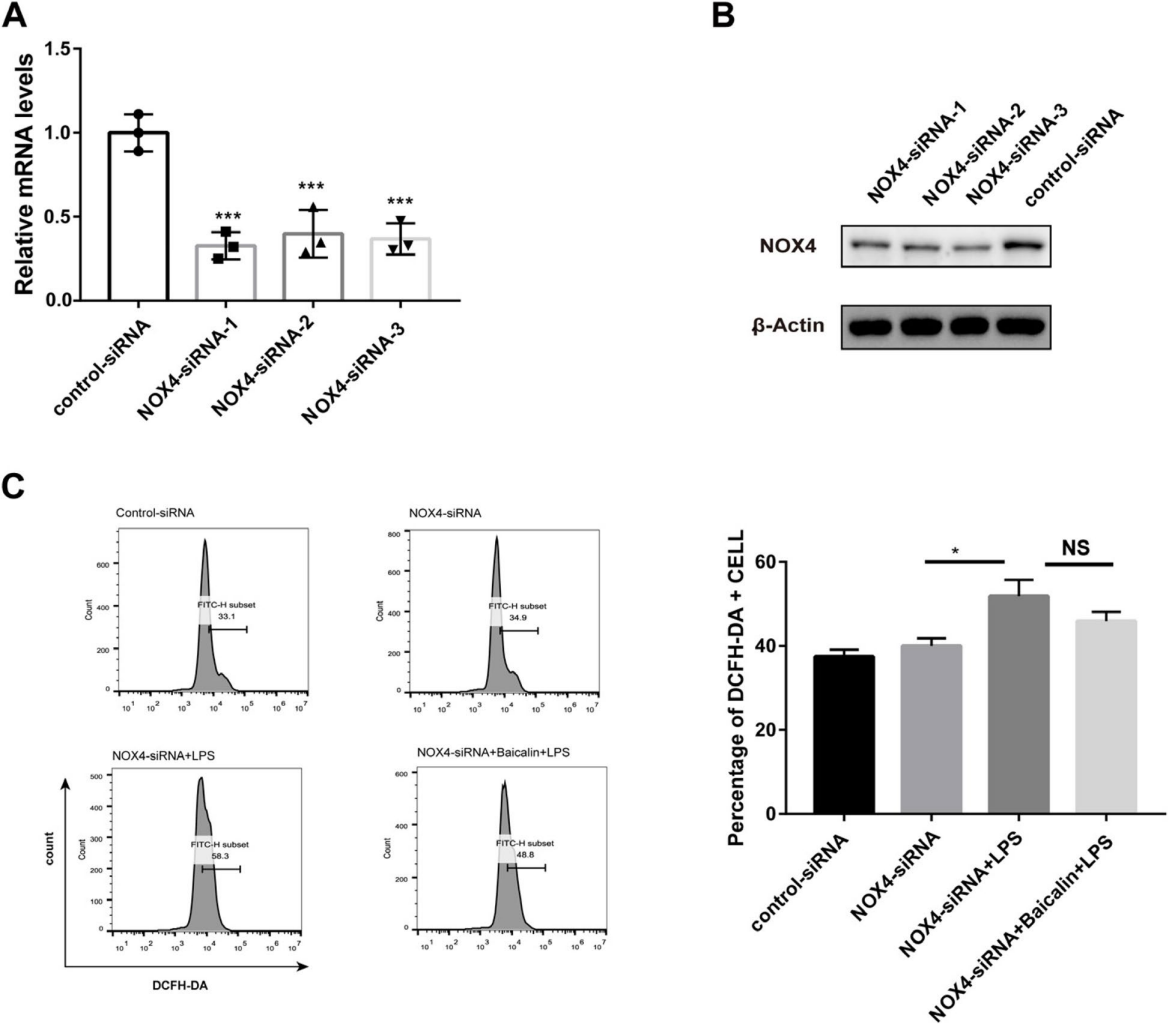
Low expression of NXO4 attenuated the inhibition of LPS-induced ROS production by baicalin. **A** NOX4 mRNA expression in HAECs infected with control-siRNA, NOX4-siRNA-1, NOX4-siRNA-2 and NOX4-siRNA-3. All NOX4-siRNAs remarkably downregulated NOX4 mRNA. **B** Western blotting analysis of the expression of NOX4 after the suppression of NOX4 expression. **C** The expression of ROS content in HAECs transfected with NOX4-siRNA or control-siRNA after stimulation with or without LPS (1 μg/ml). ROS levels were detected by flow cytometry analysis. Data were shown as mean ± SEM. (*n* = 3, *P < 0.05, ***P < 0.001)

To further confirm whether the anti-oxidative stress effects of baicalin was regulated by NOX4, three pairs of NOX4 siRNAs were separately transfected into HAECs to knock down endogenous NOX4. The reduction of NOX4 mRNA was 67.24%, 60.04% and 63.14% after transfection with siRNA-1, siRNA-2 or siRNA-3, respectively (Fig. 7A). Since NOX4 protein expression was significantly weaker in HAECs transduced with siRNA-3, we selected siRNA-3 for follow-up experiments (Fig. 7B). As shown in Fig. 7C, the ROS content in NOX4-siRNA did not change significantly compared to the control-siRNA. After LPS stimulation, ROS levels were significantly higher in the NOX4-siRNA group whereas the baicalin slightly reduced ROS but not statistically (*p* = 0.2647).

## Discussion

Chinese medicine is widely used in clinical treatment in Asia. It has gradually shown its advantages owing to multi-target characteristics and positive therapeutic effects. However, the application of TCM is obstructed by its complex composition and unrecognized therapeutic mechanisms. With the flourishing development of bioinformatics and network pharmacology, it is possible to elucidate active ingredients and pharmacological mechanisms of Chinese herbal medicine. The preventive and therapeutic effects of herbs, such as Salvia miltiorrhiza, Rhizoma chuanxiong and Scutellaria baicalensis, on AS have been widely confirmed [43–45]. Scutellaria baicalensis has immunomodulatory effects on macrophages and dendritic cells, regulating the shift in cell polarization toward anti-inflammation and producing a preventive effect on foam cell formation, thereby inhibiting the development of AS [46]. Baicalin is a flavonoid derived from Scutellaria baicalensis Georgi and has antibacterial, anti-inflammatory, antioxidant, anticancer, and other functions [47, 48]. Li et al. [49] observed that baicalin inhibited cigarette smoke-induced inflammation through suppressing the phosphorylation of HDAC2. Sun et al. [50] found that baicalin could reduce the lipopolysaccharide (LPS)-induced periodontitis in rats by curbing the TLR/MYD88/NF-κB signaling pathway. However, the target genes and mechanisms of baicalin in alleviating oxidative stress and thus anti-AS remains unclear.

Oxidative modifications of lipids have been detected in vascular lesions and the degree of oxidation positively correlates with the severity of disease, suggesting a role of oxidative stress in AS [51], while the NADPH oxidase (NOX) family is an important mediator of ROS production in the vascular wall. NOX1 and NOX2 have been shown to be deleterious to vascular disease development, particularly in the context of AS. In contrast, NOX4, the major NADPH oxidase in endothelial cells, has been shown to protect against AS in several mouse models [52–54]. NOX4 mainly releases H2O2 to stimulate the expression of NOS and the formation of NO, which inhibits oxidative stress by scavenging oxygen free radicals [55, 56]. Schürmann et al. demonstrated that NOX4 is an endogenous anti-atherosclerotic enzyme and that genetic deletion of NOX4 accelerates the development of atherosclerosis in ApoE-/- mice. NOX4 deficiency in endothelial cells increases leukocyte adhesion and exacerbates endothelial inflammatory activation [57]. eNOS promotes the formation of NO in the vascular endothelium. ROS are spatially close to eNOS and can directly provoke the uncoupling of eNOS, reducing NO bioavailability and activating other redox-sensitive systems [58]. Oxidative stress was considered as the major contributor to eNOS uncoupling and endothelial dysfunction. It was also known that ROS impair endothelial NO-mediated coronary micro-vessel dilation [59].

In our study, the core gene of baicalin-AS targets was identified through network pharmacology, molecular docking, and microarray dataset analysis. As a result, NOX4 was shown to be down-regulated under endothelial damage conditions. In addition, functional enrichment analysis showed that baicalin-AS targets were associated with inflammation and oxidative stress pathways, suggesting that baicalin may act against AS via anti-inflammatory and anti-oxidative stress effects. Through literatures, we found that NOX4 was closely associated with oxidative stress. To further investigate whether baicalin is associated with reducing oxidative stress by targeting NOX4, we conducted HAECs-based molecular experiments, including RT-qPCR, western blot and flow cytometry. Interestingly, we found that baicalin elevated the expression of NOX4 and eNOS, and reduced intracellular ROS levels in LPS-induced endothelial injury model. Nevertheless, the inhibitory effect of baicalin on ROS production was diminished after down-regulation of NOX4 expression. Consequently, we conclude that baicalin may exert its pharmacological function by targeting NOX4 to alleviate oxidative stress.

Some limitations of this study should be noted. First, three different databases were used in order to obtain adequate targets for baicalin. However, some bias may exist due to the different computer algorithms used in each database. Secondly, our conclusions were only validated on one kind of endothelial cells and not tested on other endothelial cells and animals. In the next studies, other types of endothelial cells and Nox4^−/−^ mice should be added to scientifically and rigorously demonstrate the in-depth mechanism of baicalin in the alleviation of AS.

## Conclusion

Collectively, we combined network pharmacology and endothelial injury models to investigate the anti-AS mechanism of baicalin. The results demonstrate that baicalin may exert anti-oxidative stress effects by targeting NOX4, providing new mechanisms and insights to baicalin for the treatment of AS.

## Supplementary Information

**Additional file 1. **

**Additional file 2. **

## Data Availability

The dataset analyzed during the current study are available in the GEO repository, GSE132651. Other data used to support the findings of this study are available from the corresponding author upon request.
