# Supplementary material for: Exploring the protective mechanism of baicalin in treatment of atherosclerosis using endothelial cells deregulation model and network pharmacology
Source: BMC Complement Med Ther. 2022 Oct 3;22:257. doi: 10.1186/s12906-022-03738-3 (PMC9527735; doi:10.1186/s12906-022-03738-3)
Supplement: Supplementary file 2 — Additional file 2. [file 12906_2022_3738_MOESM2_ESM.zip › Raw data of western blot.zip!/Raw data of western blot/Supplementary Figure.pdf]

Raw data of Western blot

We guarante that all protein blots were obtained from the same gels.
In the experiment, we first obtained background images of uncropped PVDF membranes. Then the membranes were clipped into bands of corresponding molecular weight of proteins prior to hybridization with antibodies. At the end of the exposure, cropped bands were grouped together.
Here, we show the original images of all blots.

Figure 8C

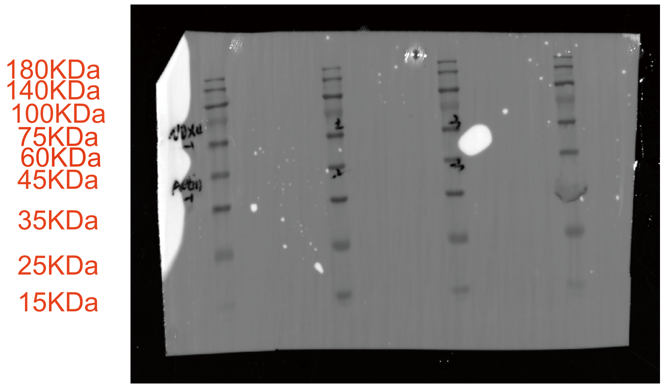

Whole membrane

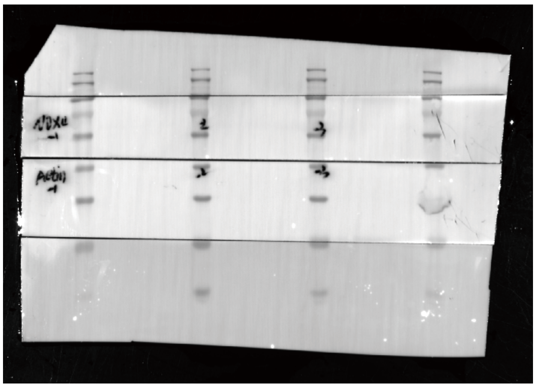

Whole membrane (cropped)

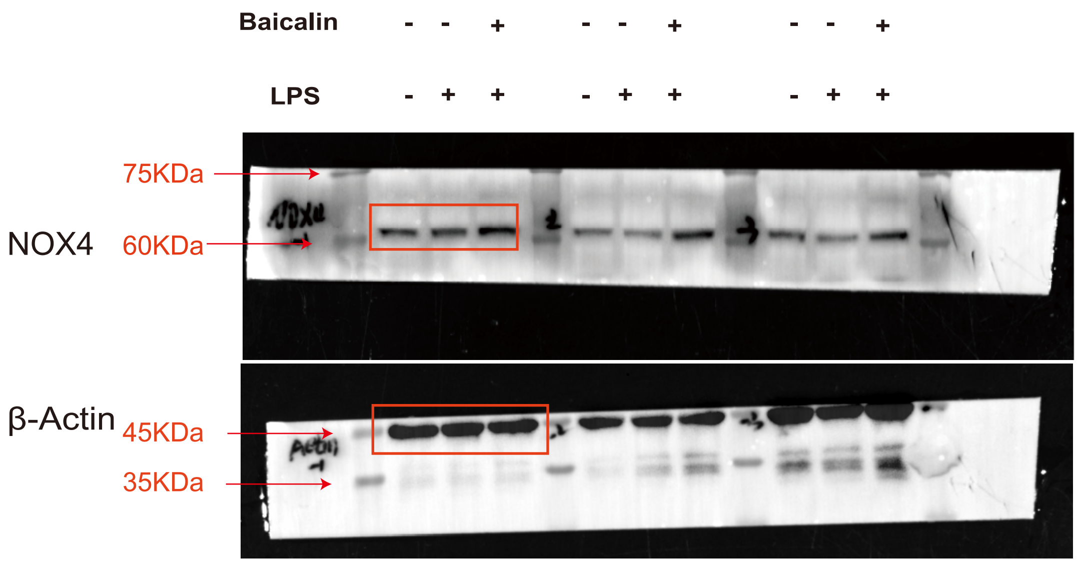

Supplementary Figure 1: Baicalin increases the protein expression level of NOX4
A. The red arrow indicates the marker site (RM19001, ABclonal)
B. The red box represents the cropped images used in the main manuscript.

Figure 9B

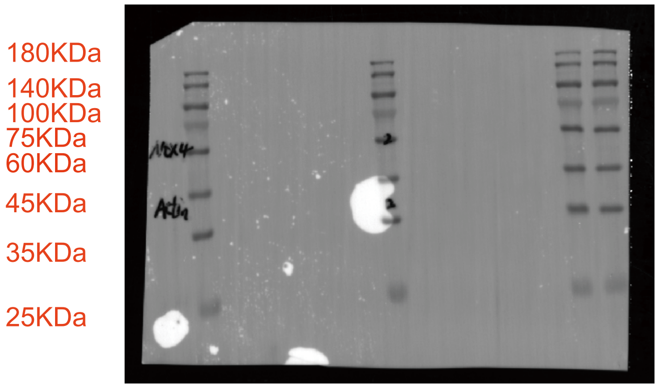

Whole membrane

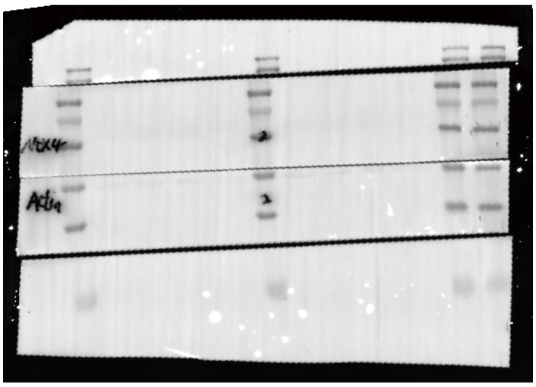

Whole membrane (cropped)

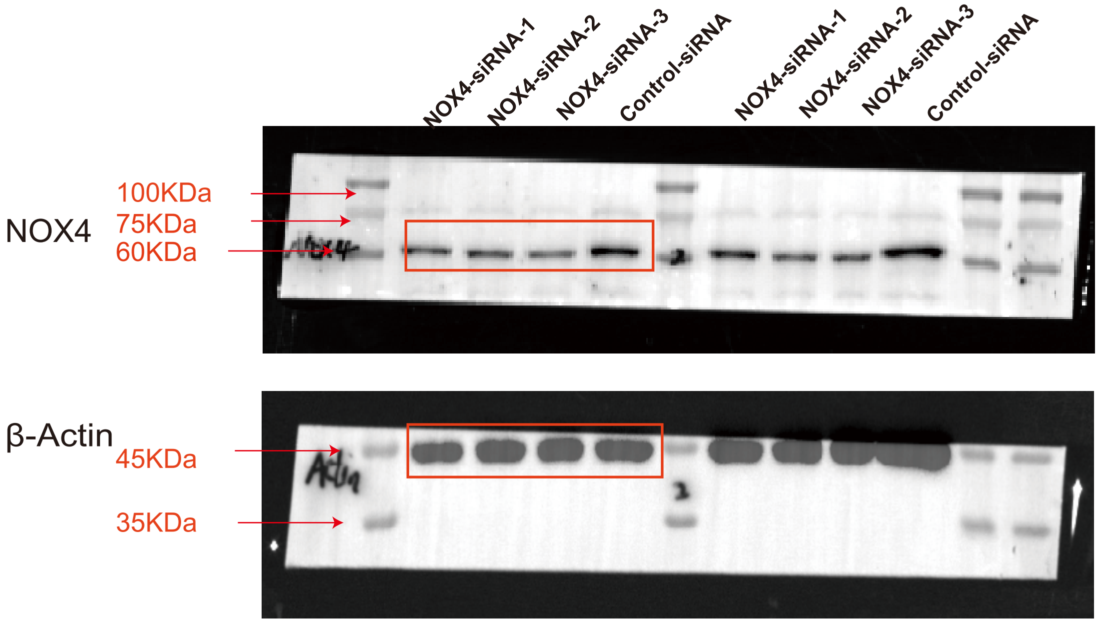

Supplementary Figure 2: Western blot analysis of NOX4 expression following suppression of NOX4 by siRNA
A. The red arrow indicates the marker site (RM19001, ABclonal)
B. The red box represents the cropped images used in the main manuscript.
